# Reviewers

**Published:** 2010-01

The journal wishes to thank the following individuals who have reviewed manuscripts during the past year. We thank you for your continued support.

Abad-Franch, Fernando

Abeyewickreme, Dr Wimal

Abraham, David

Achee, Nicole

Acosta, Luz

Adam, Rod

Afonso, Luis Carlos

Aguilar, Patricia V.

Ajjampur, Sitara Swarna Rao

Akhan, Okan

Albonico, Marco

Albright, Julia W.

Aldstadt, Jared

Al-Hasan, Majdi

Ali, Mohammed

Almeida, Paulo

Alonso, Pedro L.

Alphey, Luke

Altcheh, Jaime

Alto, Barry W.

Alvarado-Esquivel, Cosme

Alves, Carlos Roberto

Alves, Venancio

Anderson, Daniel

Andrade, Heitor Franco de

Andrade-Narvaez, Fernando J.

Andreadis, Theodore

Anisimov, Andrey P.

Ansdell, Vernon

Anstead, Greg

Anstey, Nicholas

Antinori, Spinello

Antonini, Pietro Giuseppe

Aoun, Karim

Apperson, Charles S.

Apt, Werner B.

Arevalo, Jorge

Ari, Mari D.

Ariaratnam, Ariaranee

Arias, Mayra S.

Arnold, Ben

Aronson, Naomi

Arrowood, Michael J.

Artsob, Harvey

Aultman, Kate

Avila-Campos, Mario Julio

Ayles, Helen

Azad, Abdu F.

Babiker, Hamza Ali

Babu, Subash

Bacon, David J.

Bafende, Eric aombe

Baird, J. Kevin

Barat, Lawrence

Barcus, Mazie J.

Barennes, hubert M.

Barker, Christopher M.

Barker, Robert H.

Barnard, Donald

Barnett, Elizabeth

Barnwell, John W.

Barquez, Ruben M.

Barral-Netto, Manoel

Barrera, Roberto

Barrett, Alan David

Barrio, Alejandra Beatriz

Barroso, Paola A.

Barton Behravesh, Casey

Bartram, Jamie

Basco, Leonardo K.

Basombrio, Miguel Angel

Bassili, Amal

Bausch, Daniel

Beard, Charles Ben

Beare, Nicholas A.V.

Beasley, David

Beatty, Mark

Beck, Eduard

Becker, Norbert

Becker-Dreps, Sylvia

Beg, Mohammad A.

Behrens, Ronald H.

Beier, John C.

Belizario, Vincente

Benedict, Mark Q.

Berger, Franck

Bergmann-Leitner, Elke S.

Berman, Jonathan

Bern, Caryn

Bhattacharya, Sujit Kumar

Bhattacharya, Amit

Bhutta, Zulfiqar Ahmed

Biggs, Beverley-Ann

Bilate, Angelina

Black, William Cormack

Black, Robert E.

Blacksell, Stuart D.

Blair, Carol D.

Blair, Patrick James

Blanchard, Daniel

Blitvich, Bradley

Bockarie, Moses John

Bodeker, Gerard

Boiron, Patrick

Bosch, Irene

Bosio, Christopher F.

Bottiger, Blenda

Boughey, Simon

Boulouis, Henri-jean

Bouyer, Donald

Bowen, Richard A.

Bowman, Dwight Douglas

Branch, Oralee

Brandling-Bennett, A. David

Brault, Aaron C.

Bray, Mike

Breiman, Robert F.

Bretagne, Stephane

Brogdon, William

Brooker, Simon

Brouqui, Philippe

Broussard, Cheryl

Brown, Charles

Brown, Heidi E.

Brown, Joseph Mark

Brubaker, Robert R.

Bruckner, David A.

Brun, Reto

Brunetti, Enrico

Brustoski, Kim

Bryant, Juliet E.

Buchy, Philippe

Buck, Gregory A.

Buffington, Joanna

Bulla, Lee

Buller, Mark

Calo Romero, Eliete

Cama, Vitaliano

Campbell, Andrew G.

Campbell, David A.

Campbell, Karen

Cappa, Stella Maris Gonzalez

Cappello, Michael

Carlier, Yves

Carrington, Christine V.F.

Casanova, Lisa

Castelli, Fracesco

Castilho, Euclides Ayres

Catteruccia, Flaminia

Caumes, Eric

Causer, Louise

Certad, Gabriela

Chacin-Bonilla, Leonor

Chadee, Dave D.

Chang, FY

Chang, KP

Chang, Kwang-Poo

Chappuis, Francois

Chaves, Luis Fernando

Cheever, Allen W.

Chen, Grace L.

Chen, Lin H.

Chen, Steve S.-L.

Cheng, Allen C.

Cheng, Qin

Chetchotisakd, Ploenchan

Chippaux, Jean-Philippe

Chomel, Bruno B.

Chulay, Jeffrey D.

Chung, Doo Ryeon

Ciftci, Ergin

Clark, J. Marshall

Clennon, Julie A.

Coffey, Lark L.

Coldren, Rodney L.

Colford, John M.

Colley, Dan

Comer, James A.

Confalonieri, Ulisses

Conn, Jan

Consigny, Paul Henri

Cook, Joseph

Cooper, Philip

Cordeiro da Silva, Anabela

Cornel, Anton J.

Counihan, Helen

Cox, Sharon

Coyne, Philip

Craig, Peter

Cross, John H.

Culleton, Richard Leighton

Curtis, Val

Cusick, Sarah Eastman

da Silva, Antonio Menezes

Dabritz, Haydee Ann

Daily, Johanna P.

Dalton, John P.

Dance, David

Dantas-Torres, Filipe

Dasch, Gregory A.

Dash, Paban

Daugherty, Jon R.

Davaalkham, Dambadarjaa

David, John R.

Davies, F. Glyn

Davis, Jennifer

Day, Jonathan F.

Day, Nick

de Castro, Solange

de la Fuente, Jose

de Lamballerie, Xavier

de Lemos, Elba R. S.

de Mast, Quirijn

De Sousa, Rita

Deardorff, Eleanor Rose

Deborggraeve, Stijn

Dedet, Jean-Pierre

Dees, William

della Torre, Alessandra

Deloron, Philippe

Depaquit, Jerome

Dessein, Alain J.

Dewey, Cate

Diallo, Mawlouth

Diallo, Mamadou

Dib, Juan Carlos

Didier, Elizabeth

Diemert, David J.

Dobson, Stephen

Doherty, Tom

Dondorp, Arjen M.

Donelly, Martin

Dong, John Y.

Donnelly, Martin

dos-Santos, Washington L. C.

Drebot, Mike

Drekonja, Dimitri

Druilhe, Pierre

Du Preez, Martella

Dube, Anuradha

Dupont, Herbert L.

Dysko, Robert C.

Eckmann, Lars

Edelman, Robert

Effler, Paul V.

Eggers, Jeffrey

Eisen, Rebecca J.

Eisen, Lars

El Sherbini, Azza A.

Elder, John P.

Elliott, Mark

Ellis, Brett Richard

Ellis, Alicia

Endy, Timothy P.

Engel, Amber

Engelthaler, David M.

Engman, David M.

Enria, Delia

Enscore, Russell E.

Enwonwu, Cyril O.

Erikstrup, Christian

Espinoza, Bertha

Estep, Scot

Evans, Carlton

Facchinelli, Luca

Fahal, Ahmed Hassan

Fahrmeir, Ludwig

Failloux, Anna-Bella

Faix, Dennis

Fan, CK

Fasel, Nicolas

Fayer, Ronald

Feilij, Hector

Feliciangeli, M. Dora

Fernandez-Ibanez, Pilar

Ferreira, Marcelo Urbano

Field, Linda

Fierer, Joshua

Fischer, Egil Andreas Joor

Fischer, Peter

Fischer, Philip R.

Flisser, Ana

Floeter-Winter, Lucile

Foley, Janet E.

Fonseca, Dina M.

Forshey, Brett

Fournier, Pierre-Edouard

Fowkes, Freya

Foy, Brian

Freedman, David O.

Freier, Jerome E.

Furusyo, Norihiro

Gage, Kenneth L.

Gajadhar, Alvin

Galloway, Renee

Gangarosa, Eugene

Ganta, Roman

Garba, Amadou

Garcia, Hector H.

Garcia, Lynne

Garg, Nisha Jain

Gassama-Sow, Amy

Gee, Jay E.

Ghosh, Susanta Kumar

Gibbons, Robert V.

Gillin, Frances D.

Gilman, Robert H.

Gimnig, John E.

Giron, Jose Antonio

Githeko, Andrew

Glass, Mindy Buie

Glass, Gregory E.

Gobert, Geoff

Goldblatt, David

Goncalves, Denise

Gonzalez Cappa, Stella

Gonzalez, Armando E.

Goodman, Catherine A.

Goris, Marga

Gottstein, Bruno

Gould, Fred

Grab, Dennis

Gradoni, Luigi

Graeff-Teixeira, Carlos

Granger, Donald L.

Granwehr, Bruno Palma

Graves, Stephen

Green, Sharone

Greenwood, Brian

Griffin, Diane

Grillet, Maria Eugenia

Gromowski, Greg

Grubhoffer, Libor

Gschwend, Phil

Gu, Weidong

Gubler, Duane J.

Guerra, Marta A.

Guerra, Humberto 

Guerrant, Richard

Guffanti, Monica

Guo, How-Ran

Gurtler, Ricardo E.

Halstead, Scott B.

Hamburger, Joseph

Hamer, Davidson

Hamer, Gabriel Lee

Hanley, Kathryn Alyce

Happi, Christian

Harley, David O.

Harrington, Laura C.

Harrus, Shimon

Harth, Guenter

Harvey, Steven A.

Haselow, Nancy J.

Hayes, Edward B.

Henriquez-Camacho, Cesar AJ

Herrera, Socrates

Heye, Tobias

Higgs, Stephen

Hise, Amy

Hoffman, Stephen L.

Holbrook, Mike

Holland, Celia Victoria

Holmes, Eddie C.

Hoque, Bilqis Amin

Horii, Toshihiro

Horton, John

Hung, Yao-Min

Hunsperger, Elizabeth

Huston, Christopher D.

Ibarra, Humberto

Idro, Richard

Igarashi, Ikuo

Irby, William S.

Ishikawa, Tomohiro

Ismail, Nahed

Ito, Akira

Jacob, Benjamin G.

Jacobsen, Kathryn H.

Jacobson, Raymond L.

Jaffe, Charles

Jansen, Ana Maria

Jarman, Richard G.

Jelinek, Tomas

Jia, Wanzhong

Jilcott, Stephanie

Jin, Xia

Johnson, Barbara W.

Johnson, Mary Beth

Johnston, Richard B.

Jones, Jeffrey L.

Jonsson, Colleen B.

Joshi, Hema

Joy, Deirdre

Joyce, M. Patricia

Judah, Gaby

Kachur, S. Patrick

Kager, Piet A.

Kain, Kevin

Kalayanarooj, Siripen

Kamhawi, Shaden

Kaneko, Osamu

Kaplan, Gilla

karunajeewa, Harin

Kato-Hyashi, Naoko

Katz, Alan R.

Katz, Michael

Kaye, Paul

Kayentao, Kassoum

Kazanjian, Powel

Keating, Joseph

Kelly, Daryl J.

Kelly, Paul

Kent, Rebekah

Keysary, Avi

khatab, khaled

Kilpatrick, A Marm

Kim, Dong-Min

King, Charles H.

Kirchhoff, Louis V.

Kirk, Gregory

Kishimoto, Miori

Kiszewski, Anthony

Kitron, Uriel

Kleinschmidt, Immo

Klion, Amy D.

Klotz, Stephen A.

Knols, Bart

Kochel, Tadeusz

Koenraadt, Sander

Kollars, Tom

Koram, Kwadwo A.

Kosek, Margaret N.

Kosoy, Michael

Kothera, Linda

Kramer, Laura D.

Krishna, Sanjeev

Krishnan, Triveni

Krolewiecki, Alejandro Javier

Ksiazek, Thomas G.

Kumwenda, N.

Kurtis, Jonathan D.

Labruna, Marcelo B.

Lambrechts, Louis

Lammie, Patrick J.

Lana, Marta

Lane, Joshua E.

Lane, Robert S.

Laufer, Miriam

Lazzari, Claudio Ricardo

Leake, John

LeDuc, James W.

Lee Ho, Paulo

Lee, Chao-Hung

Lefrancois, Thierry

Leiby, David A.

Leisnham, Paul

Lengeler, Christian

Lenhart, Audrey

Leontsini, Elli

Letonja, Thomas

Levett, Paul N.

Levy, Michael Z.

Lewallen, Susan

Liang, Song

Libraty, Daniel H.

Lima, Marli Maria

Limmathurotsakul, Direk

Lin, Feng Ying C.

Lindegardh, Niklas

Lindler, Luther

Lindsay, Steven W.

Linthicum, Kenneth J.

Llanos-Cuentas, Alejandro

Lloyd, Linda

Lockwood, Diana N. J.

Long, Maureen T.

Lorca, Myriam

Lord, Cynthia

Loscher, Thomas

Lotufo, Paulo A.

Love, David

LoVerde, Philip T.

Lu, Shan

Lubell, Yoel

Luby, Stephen P.

Lule, John Ronald

Lushbaugh, William B.

Luz, Paula Mendes

Lyke, Kirsten

Maciel, Ethel

MacKenzie, Charles

Maco, Vicente

Magez, Stefan

Magill, Alan J.

Magnaval, Jean-Francois

Magori, Krisztian

Maguire, James H.

Maguire, Jason D.

Mahajan, Sanjay K.

Makame, Ame Shaali

Makler, Michael T.

Maleewong, Wanchai

Manda, Samuel O. M.

Manguin, Sylvie

Maniania, Nguya K.

Mansuy, Jean-Michel

Marcos, Luis Augusto

Marovich, Mary A.

Marques, Ernesto Torres de Azevedo

Marra, Christina

Martin, Diana

Masuoka, Penny

Mathew, Anuja

Maude, Richard James

Mauricio, Isabel

Maurin, Max

McBride, Jere

McCarthy, James S.

McCubbin, Colin

McCutchan, Allan

McDevitt, Michael

McDowell, Mary Ann

McElroy, Peter D.

McFadden, Grant

McGarvey, Stephen

McGwire, Bradford S.

McLean, Robert G.

McLellan, Susan L. F.

McLeod, Anni

McQuiston, Jennifer

Mehta, Sanjay Ramesh

Mendez, Ignacio

Meshnick, Steve

Meyerhoff, Andrea

Meyers, Wayne M.

Michael, Edwin

Michel, Andy P.

Milhous, Wilbur K.

Miller, Barry R.

Millet, Pascal G.

Mills, Edward

Mintz, Eric

Mitre, Edward

Molaei, Goudarz

Monath, Thomas P.

Moncayo, Alvaro

Monteon, Victor M.

Montgomery, Joel Mark

Moon, Roger

Moore, Julie

Moore, Thomas A.

Moorthy, Vasee

Moran, John S.

Morenikeji, Olajumoke Abimbola

Moreno, Javier

Mores, Christopher N.

Morgan, Juliette

Morita, Kouichi

Moro, Manuel H.

Morrison, Amy Celeste

Morrow, Richard H.

Moyer, Richard W.

Mu, Jianbing

Mueller, Ivo

Munayco, César Vladimir

Murdoch, David R.

Murgue, Bernadette

Murray, Kristy

Nahlen, Bernard L.

Nasci, Roger

Nash, Theodore

Neerinckx, Simon

Nelson, Kenrad E.

Nelson, Stephen

Nevin, Remington Lee

Nielsen, Carrie F.

Nieto, Nathan C.

Nisalak, Ananda

Njemanze, Philip Chidi

Noedl, Harald

Nolan, Thomas J.

Nolder, Debbie

Noor, Abdisalan

Noyes, Harry

Nutman, Thomas

Nwakanma, Davis Chibuzo

Nyunt, Myaing Myaing

Ochoa, Theresa Jean

Ofori, Owusu

Oka, T.

Okada, Kazuhisa

Okamoto, Hiroaki

Okanurak, Kamolnetr

Okeke, Iruka N.

Oldfield, Edward C.

Olliaro, Piero

Olusanya, Bolajoko

Oteo, José  Antonio

Oundo, Joseph Owiti

Owen, Jennifer

Paessler, Slobodan

Panzera, Francisco

Paris, Daniel Henry

Paskewitz, Susan

Pasvol, Geoffrey

Paz-Soldan, Valerie

Pearson, Richard D.

Pelloux, H.

Pennington, Pamela Marie

Pereira, Daniela Pita

Pereira, Martha Maria

Pereira, ricardo

Perkins, Peter V.

Perrin, Mercio

Petersen, Christine

Petersen, Lyle

Peterson, David

Petri, William

Petti, Cathy

Pfarr, Kenneth

Picot, Stephane

Piesman, Joseph

Piyaphanee, Watcharapong

Portus, Montserrat

Postan, Miriam

Powell, Craig

Powell, Jeffrey

Powers, Ann M.

Prakash, John

Price, Lance

Price, Ric

Price, Richard

Prince, Harry

Psaroulaki, Anna

Purcell, Kevin

Putnak, Joseph Robert

Quinnell, Rupert

Rabello, Ana

Rainey, Petrie M.

Rainville, Alice Jo

Ram, Pavani

Ramos, Mary M.

Ranson, Hilary

Rao, Ramakrishna U.

Raoult, Didier

Ravel, Christophe

Ready, Paul

Reed, Sharon

Reisen, William K.

Reisen, William K.

Reithinger, Richard

Restrepo, Blanca

Reyburn, Hugh

Reynolds, Steven J.

Ribolla, Paulo

Richards, Allen L.

Richards, Stephanie Lynn

Richie, Thomas L.

Richter, Dania

Riera, Cristina

Rikihisa, Yasuko

Ringwald, Pascal

Ritchie, Scott Alex

Ritmeijer, Koert

Roberts, Donald R.

Rodriguez, Americo

Rodriguez-Iglesias, Manuel

Rodriguez-Morales, Alfonso J.

Rodriguez-Perez, Mario

Rodwell, Timothy

Roeder, Peter Leonard

Roehrig, John T.

Rogers, William O.

Rogerson, Stephen J.

Rogier, Christophe C.

Rolain, Jean-Marc

Romig, Thomas

Rosenthal, Philip J.

Rothman, Alan

Rowe, Alexander

Rowland, Mark

Rushton, Jonathan

Ruslami, Rovina

Russell, Bruce M.

Ryan, Peter A.

Saathoff, Elmar

Sack, David

Sack, R. Bradley

Sacks, David

Saha, Debasish

Sahni, Sanjeev

Sako, Yasuhito

Saleh, Maya

Salkeld, Daniel

Salomon, Daniel

Samuelson, John

Sankara, Dieudonne Pabeyba

Santiago, Helton

Santos-Ciminera, Patricia Dantas

Saraswathy, T.S.

Saravia, Nancy Gore

Savage, Harry M.

Sawanyawisuth, Kittisak

Schantz, Peter M.

Scheel, Christina M.

Schijman, Alejandro G.

Schlesinger, Jacob J.

Schmidt, Wolf-Peter

Schnaufer, Achim

Schoenian, Gabriele

Scholle, Frank

Schwab, Kellogg

Scollard, David

Scott, Alan L.

Scott, Anthony

Secor, William Evan

Semnani, Roshanak Toulouei

Sethi, Nitin K.

Severson, David

Seybolt, Lorna

Shanks, G Dennis

Shanks, G. Dennis

Shapiro, H. M.

Sharma, Yagya

Shaw, Jeffrey

Shiff, Clive

Shillcutt, Samuel Dedman

Shin, Sonya

Sibley, Carol Hopkins

Silber, Ariel Mariano

Silveira, Thais Gomes Verzignassi

Silvestre, Ricardo

Simmons, Cameron

Simondon, Kirsten

Sloan, Derek

Sloan, Jeffrey

Slotman, Michel A.

Smith, David William

Smith, Michael

Smythe, Lee

Snounou, Georges

Snow, Robert W.

Sorvillo, Frank

Sosa-Estani, Sergio

Souris, Marc

Southern, Paul M.

Spear, Robert

St. Jeor, Stephen C.

Stauber, Christine E.

Stauber, Rudolf

Stauffer, William

Steketee, Richard W.

Stenseth, Nils C.

Stergachis, Andy

Stewart, Ann V.

Stiles, Jonathan K.

Stinchcomb, Dan

Stoddard, Steven T.

Strickland, George Thomas

Strickman, Daniel

Stump, Aram

Suaya, Jose A.

Subhadra, Nandakumar

Sullivan, David Joseph

Suputtamongkol, Yupin

Svoboda, Michal

Syafruddin, Din

Tanabe, Kazuyuki

Tanowitz, Herbert B.

Tapia, Felix J.

Targett, Geoffrey

Tatem, Andrew J.

Taylor, Bob R. J.

Taylor, Charles

Taylor, David

Taylor, Terrie E.

Teixeira, Maria Jania

Telford, Sam R.

Temu, Emmanuel A.

Tesh, Robert

Teunis, Peter

Thammapalerd, Nitaya

Thomas, Stephen J.

Thwing, Julie

Tibayrenc, Michel

Todd, Charles W.

Tomashek, Kay M.

Torrico, Faustino

Traub-Cseko, Yara Maria

Travi, Bruno L.

Triana, Omar

Truman, Richard W.

Tsetsarkin, Konstantin

Tsuboi, Takafumi

Turell, Michael J.

Tyler, Kevin

Ubol, Sukathida

Unnasch, Thomas R.

Unsworth, Nathan B.

Valecha, Neena

Valerio, Laura

van der Werf, Tjip S.

Van Nassauw, Luc J. P.

Vanden Eng, Jodi

Vanlerberghe, Veerle

Varki, Nissi

Vasilakis, Nikolaos

Vaughan, Jefferson A.

Vazquez, Gonzalo

Veronese, Francisco

Verra, Federica

Vinetz, Joseph M.

Virreira, Myrna

Volk, Sara

Walker, David H.

Walker, Ned

Walsh, Douglas S.

Wang, Changlu

Wanji, Samuel

Ward, Honorine D.

Watt, George

Watts, Douglas

Weedall, Gareth

Wei-Gang, Qui

Weina, Peter J.

Wenger, Jay

Werneck, Guilherme L.

Wernsdorfer, Walter H.

Wernsdorfer, Walther Helmut

Wex, Thomas

Whary, Mark

White, A. Clinton

White, Nicholas

Wikel, Stephen

Wilkerson, Richard C.

Wilkins, Patricia P.

Williams, Holly Ann

Wills, Bridget

Wilson, Mary E.

Wilson, William C.

Winzeler, Elizabeth

Wong, Susan J.

Wongsrichanalai, Chansuda

Woodall, John P.

Woods, Christopher W.

Woods, Michael Edward

Wormser, Gary P.

Wortmann, Glenn

Wright, James

Yabsley, Michael J.

Yamaoka, Yoshio

Yardley, Vanessa

Yazdanbakhsh, Maria

Yeh, Trai-Ming

yibiao, zhou

Yoksan, Sutee

Yoon, In Kyu

Young, Ed

Yuill, Thomas M.

Yukich, Joshua

Zahn, Manuel

Zavala-Castro, Jorge

Zeledon, Rodrigo 

Zhou, Xiao-Nong

Zhou, Guofa

Zicker, Fabio

Zimmerman, Peter A.

Zurovac, Dejan

